# Gas-Sensing Properties of B/N-Modified SnS_2_ Monolayer to Greenhouse Gases (NH_3_, Cl_2_, and C_2_H_2_)

**DOI:** 10.3390/ma15155152

**Published:** 2022-07-25

**Authors:** Aijuan Zhang, Aijuan Dong, Yingang Gui

**Affiliations:** 1 College of Physics and Electronic Engineering, Xianyang Normal University, Xianyang 712000, China; zhangaijuan2019@163.com; 2 Qinhuangdao Vocational and Technical College, Qinhuangdao 066100, China; 3 College of Engineering and Technology, Southwest University, Chongqing 400715, China; yinganggui@swu.edu.cn

**Keywords:** greenhouse gases, SnS_2_, surface modification, adsorption, DFT

## Abstract

The adsorption capacity of intrinsic SnS_2_ to NH_3_, Cl_2_ and C_2_H_2_ is very weak. However, non-metallic elements B and N have strong chemical activity, which can significantly improve the conductivity and gas sensitivity of SnS_2_. Based on density functional theory, SnS_2_ was modified with B and N atoms to analyze its adsorption mechanism and gas sensitivity for NH_3_, Cl_2_ and C_2_H_2_ gases. The optimal structure, adsorption energy, state density and frontier molecular orbital theory are analyzed, and the results are in good agreement with the experimental results. The results show that the adsorption of gas molecules is exothermic and spontaneous. Only the adsorption of NH_3_ and Cl_2_ on B-SnS_2_ belongs to chemical adsorption, whereas other gas adsorption systems belong to physical adsorption. Moderate adsorption distance, large adsorption energy, charge transfer and frontier molecular orbital analysis show that gas adsorption leads to the change of the conductivity of the modified SnS_2_ system. The adsorption capacity of B-SnS_2_ to these gases is Cl_2_ > NH_3_ > C_2_H_2_. The adsorption capacity of N-SnS_2_ is NH_3_ > C_2_H_2_ > Cl_2_. Therefore, according to different conductivity changes, B-SnS_2_ and N-SnS_2_ materials can be developed for greenhouse gas detection of gas sensors.

## 1. Introduction

With the continuous progress of society, the traditional agricultural production mode has been unable to meet the needs of modern civilization, which prompts the development of greenhouse planting [1,2,3]. It can be applied in the plateau, deep mountains, deserts, and other unique environments for agricultural production [4,5]. The illuminance, temperature, humidity, and gas composition are the critical environmental parameters that affect planting growth [6]. In the actual cultivation process, the illuminance, temperature, and humidity can be easily regulated by changing the ceiling coverage and ventilation rate [7,8]. It is urgent to accurately monitor the greenhouse’s characteristic gas composition in the greenhouse online. Due to the half-open structure of the greenhouse, the accumulated gases are mainly NH_3_, Cl_2_, and C_2_H_2_ [9,10].

Two-dimensional SnS_2_ is widely used in the gas sensor industry because of its large specific surface area and pore structure [11]. Compared with carbon nanotubes, SnS_2_ is more resistant to oxidation and more stable at high temperatures, making SnS_2_ more suitable for gas-sensing detection than carbon nanotubes [12]. It has become one of the most promising materials used in high-temperature and high-pressure environments [13]. However, pristine SnS_2_ has a limited reaction to gases, such as C_2_H_4_, C_2_H_2_, and NH_3_ [14]. Studies showed that non-metals modification could improve the gas detection accuracy and adsorption capacity of gas-sensing materials by regulating their energy gap and conductivity upon gas adsorption [15]. B and N are the most widely used modified non-metals to improve the sensitivity, selectivity, and reaction recovery time of gas-sensing materials [16,17].

Based on the density functional theory (DFT) study, B, N-modified SnS_2_ is proposed as a promising sensor material for gas-sensing application in greenhouses, which can evaluate the change in concentration of NH_3_, Cl_2_, and C_2_H_2_ gases [18]. First, the most stable structure and the best modification performance of B, N-modified SnS_2_ was built and optimized [19]. Then, the most stable structure was chosen to analyze its adsorption mechanism to NH_3_, Cl_2_, and C_2_H_2_. By analyzing the structural optimization, adsorption energy, density of state (DOS), and charge transfer of gas molecules adsorption on B, N-modified SnS_2_, it is found that the modified SnS_2_ sensor shows high sensitivity to NH_3_, Cl_2_, and C_2_H_2_ [20,21]. This study provides a alternative approach for preparing SnS_2_-based gas sensors for the online monitoring of greenhouse gases [22,23,24].

## 2. Computational Details

All calculations were carried out based on DFT [23,25,26]. The SnS_2_ crystal plane is modeled with a 4 × 4 × 1 supercell [27,28,29,30]. To prevent the interaction from repeating planes along the z-axis direction, a vacuum layer of 25 Å was set between the planes [31,32]. The electron exchange and correlation energy were treated with the generalized gradient approximation (GGA) and the Perdew–Burke-Ernzerhof (PBE) basis [30]. A double numerical plus polarization (DNP) basis set was used [27]. The ionic convergence criterion for the total energy and maximum force were set as 1 × 10^−5^ Ha, and 2 × 10^−3^ Ha/Å, respectively [33,34], and the electronic self-consistent field tolerance was 1 × 10^−6^ Ha [30]. The Brillouin zone was sampled with a 5×5×1 Monkhorst-Pack mesh of k-points [33]. All calculations are performed under 0 K; the adsorption performance under room temperature is directly related to the results under 0 K.

The adsorption energy (*E*_ads_) of the molecule adsorbed on the SnS_2_ surface was calculated by *E*_ads_ = *E*_slab/gas_ − *E*_slab_ − *E*_gas_. *E*_slab/gas_ is the total energy of the adsorption system; *E*_slab_ and *E*_gas_ are the energy of the SnS_2_ surface and gas molecules of greenhouse gases, respectively [34]. A negative value of *E*_ads_ means the adsorption process is exothermic and happens spontaneously [35]. The electron density distribution was calculated by Mulliken population analysis [36]. The charge transfer *Q* in the adsorption process was obtained by *Q* = *Q*_ads_ − *Q*_iso_. *Q*_iso_ and *Q*_ads_ are the total charges of isolated gas and adsorbed gas molecules, respectively [37]. *Q* > 0 means electrons transfer from the gas molecules to the surface of SnS_2_. According to frontier molecular orbital theory, the energy gap represents the difference between the highest occupied orbital (HOMO) and the lowest occupied orbital (LUMO) [38]. The energy gap between HOMO and LUMO was defined by *E_g_ = |E_HOMO_* − *E_LUMO_|*. The smaller the energy gap is, the more efficiently the reaction is excited.

## 3. Results and Discussion

### 3.1. Geometry Optimization

**Figure 1:**
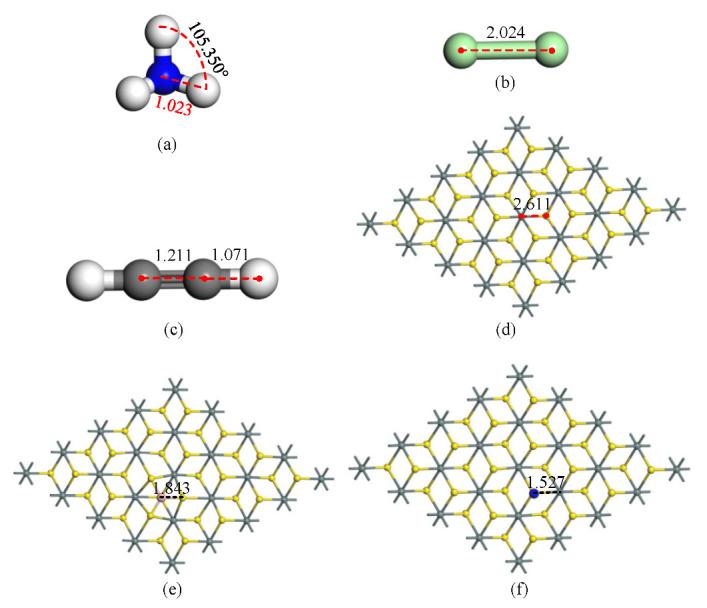
The optimized structures: (**a**) NH_3_, (**b**) Cl_2_, (**c**) C_2_H_2_, (**d**) SnS_2_, (**e**) B-SnS_2_ surface, (**f**) N-SnS_2_ surface. The distance is Å.

To obtain the adsorption characteristics of B-SnS_2_ and N-SnS_2_ to the greenhouse gases, the structures of the gas molecules and SnS_2_ surface were initially optimized. The structures of NH_3_, Cl_2_, and C_2_H_2_ gas molecules are established as shown in Figure 1a–c. The bond length of Sn-S in SnS_2_ is 2.611 Å. C_2_H_2_ gas molecule is a two-dimensional planar structure with only 1.211 Å C-C bond length and 1.071 Å C-H bond length. NH_3_ gas molecule is a regular tetrahedral structure: all of the N-H bond lengths are 1.023 Å, and the bond angles are 105.350°. Cl_2_ has a bond length of 2.024 Å. The two most stable modification structures of B and N modification on the SnS_2_ surface are obtained, respectively, as shown in Figure 1e,f. Based on the Mulliken population, B and N atoms as electron acceptors, 0.176 *e* electrons and 0.65 *e* electrons are obtained from SnS_2_. This redistribution of charge leads to a change in the system’s conductivity. It can be seen that the modification distance is 1.843 Å and 1.527 Å, respectively. From the bonding distance and charge transfer, both B and N atoms have built a stable structure on the SnS_2_ surface, which provides a foundation for further gas adsorption.

**Figure 2:**
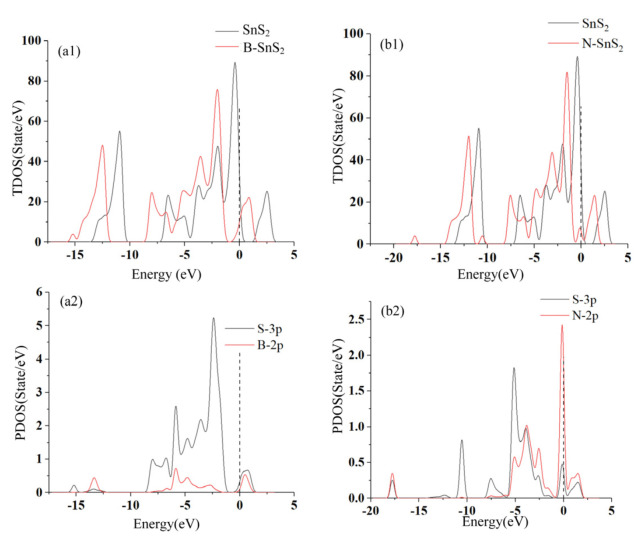
(**a1**) TDOS of SnS_2_ and B-SnS_2_, (**b1**) TDOS of SnS_2_ and N-SnS_2_, (**a2**) PDOS of B-SnS_2_, (**b2**) PDOS of N-SnS_2_.

As shown in Figure 2, the total density of states (TDOS) and partial density of states (PDOS) are analyzed to further analyze the modification mechanism of the B and N atoms on SnS_2_. Both B and N atom modifications make the TDOS move to the left. Therefore, after the modification of SnS_2_ by B and N, the electrons in the conduction band are reduced, resulting in a decrease in the conductivity of SnS_2_. According to the PDOS, the peaks of S-3*p* and B-2*p* overlap range from −6 eV to −4 eV, and at the 1 eV for the B-SnS_2_ system. On the other hand, the peaks of S-3*p* and N-2*p* hybridize around −5 eV, −4 eV, −2.5 eV, 0 eV, and 2 eV. In general, the conductivity of the modified SnS_2_ systems decreases due to the strong electronegativity of the modified atoms.

**Figure 3:**
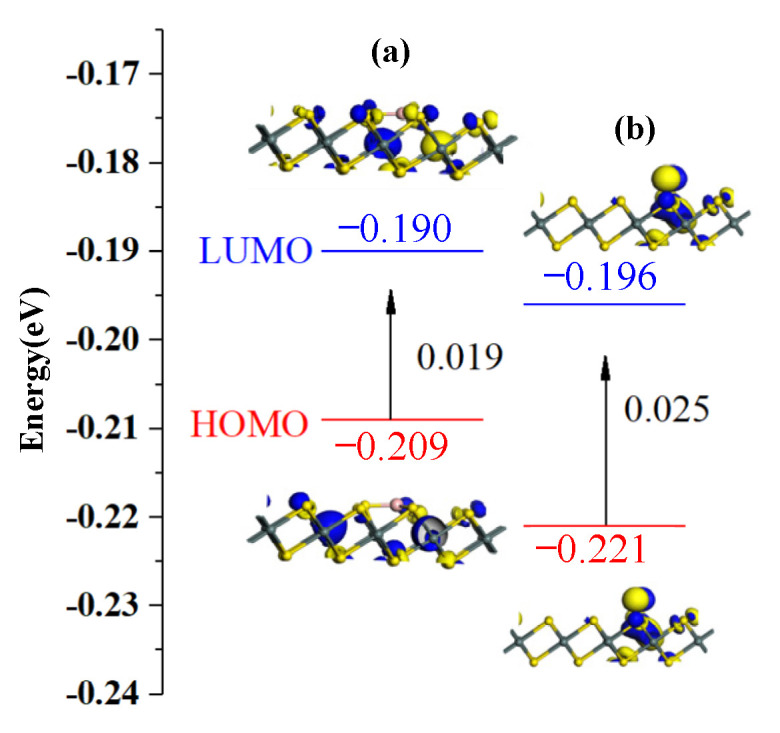
HOMO and LUMO of (**a**) B-SnS_2_ and (**b**) N-SnS_2_.

As shown in Figure 3, after B and N modification on SnS_2_, HOMO is mainly distributed on B and N, indicating that B and N atoms provide electrons as electron donors and are active sites that can provide adsorption sites for NH_3_, Cl_2_, and C_2_H_2_ gases. Moreover, the energy gap increases significantly after modification as listed in Table 1, making the system’s conductivity significantly decrease; therefore, the measurement system’s conductivity change is more pronounced. The results obtained by the frontier molecular orbital theory are consistent with those obtained by the density of state analysis.

### 3.2. NH_3_, Cl_2_, and C_2_H_2_ Adsorption on B-SnS_2_ and N-SnS_2_ Surfaces

**Figure 4:**
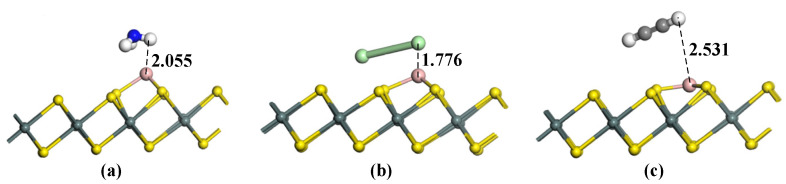
Gas adsorption on B-SnS_2_. (**a**) B-SnS_2_/NH_3_, (**b**) B-SnS_2_/Cl_2_, (**c**) B-SnS_2_/C_2_H_2_. The distance is Å.

To study the adsorption properties of the three greenhouse gases on B-SnS_2_ and N-SnS_2_, NH_3_, Cl_2_, and C_2_H_2_ gases were made to approach the B-SnS_2_ and N-SnS_2_ surfaces from different positions to obtain the most stable adsorption structures. Figure 4 shows the most stable adsorption structures after gas molecules adsorption on B-SnS_2_.

#### 3.2.1. Gas Adsorption on B-SnS_2_ Surface

Figure 4 and Table 2 show the optimal adsorption structure of NH_3_, Cl_2_, and C_2_H_2_ gas molecules on B-SnS_2_. Since the B atom is in a prominent position on the SnS_2_ surface, it provides a better attachment point for gas adsorption, making the adsorption of SnS_2_ more stable. The H and N atoms in the NH_3_ molecule were used to approach the surface of SnS_2_. The results showed that the H atom approaching the B atom method acts as the most stable structure with the largest adsorption energy (−1.735 eV). When the N atom is closed to the B atom, the adsorption energy is only −0.712 eV. The greater the absolute value of the adsorption energy, the more intense the reaction is. In addition, the negative adsorption energy means that the reaction is exothermic and can be carried out spontaneously. From the microscopic point of the adsorption structure, the bending stress also causes the surface deformation of B-SnS_2_ to different degrees. The adsorption distance of B-SnS_2_ to NH_3_ gas is 2.055 Å. The small adsorption distance indicates that the reaction may be strong chemisorption. After gas adsorption, SnS_2_ has slight deformation, and the Sn-S bond is slightly elongated. After B-SnS_2_ adsorbs NH_3_ gas, 0.254 *e* electrons transfer from the H_2_S gas to B-SnS_2_, mainly provided by the H atom. After the Cl_2_ adsorption on B-SnS_2_, the adsorption energy is −2.204 eV, the charge transfer is −0.422 *e*, and the adsorption distance is 1.776 Å. The reaction is also chemical adsorption due to the large adsorption energy and shorter adsorption distance. Upon C_2_H_2_ adsorption on B-SnS_2_, the adsorption distance is 2.531 Å, the adsorption energy is −0.272 eV, and the charge transfer is 0.172 *e*. It can be deduced that the C_2_H_2_ adsorption on B-SnS_2_ belongs to physical adsorption.

**Figure 5:**
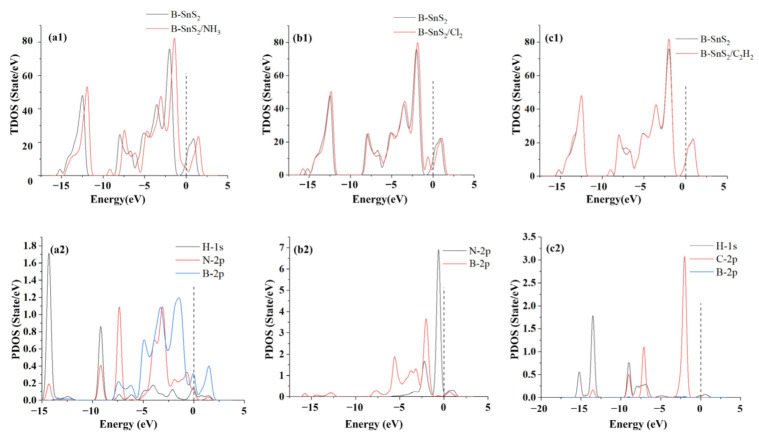
(**a1**) TDOS of B-SnS_2_ and B-SnS_2_/NH_3_, (**b1**) TDOS of B-SnS_2_ and B-SnS_2_/Cl_2_, (**c1**) TDOS of B-SnS_2_ and B-SnS_2_/C_2_H_2_, (**a2**) PDOS of B-SnS_2_/NH_3_, (**b2**) PDOS of B-SnS_2_/Cl_2_, (**c2**) PDOS of B-SnS_2_/C_2_H_2_.

Figure 5 shows the DOS analysis diagram of B-SnS_2_ after adsorption of NH_3_, Cl_2_, and C_2_H_2_; the TDOS after gas adsorption moves to the right, where the dotted line represents the Fermi energy level. From Figure 5(a1,a2), it can be figured out that the TDOS has a distinct increase above the Fermi level after NH_3_ adsorption. It facilitates the transition of electrons from the valence band to the conduction band, resulting in an overall increase in conductivity. After NH_3_ adsorption, the TDOS increases range from −5 eV to −2.5 eV, −10 eV to −12.5 eV, and 2.5 eV to 3 eV, respectively, which are caused by the hybridization of H-1*s* and B-2*p* orbitals. The strong orbital hybridization and the considerable TDOS increase indicate that this reaction is chemisorption. Additionally, the adsorption structure is very stable. When the Cl_2_ molecule is adsorbed, the TDOS of B-SnS_2_/Cl_2_ shifts to the right as a whole, and the TDOS will increase at the energy level of 0 eV, whereas the TDOS will decrease at the energy level range of −15 eV to −12 eV and −5 eV to −2 eV. The conductivity of the surface system enhances as the DOS at the Fermi level increases. The hybridization of the B-2*p* orbital and Cl-3*p* orbital shows that the reaction is very violent. According to the analysis of the DOS diagram shown in Figure 5(c1,c2), the distribution of TDOS nearly does not change before and after C_2_H_2_ adsorption.

#### 3.2.2. Gas Adsorption on N-SnS_2_ Surface

**Figure 6:**
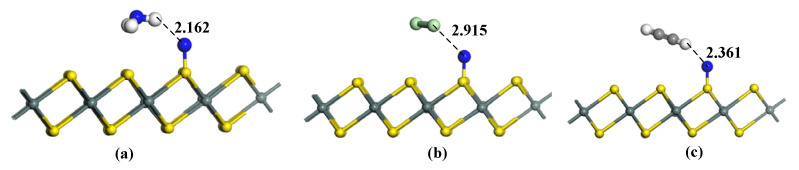
Gas adsorption on N-SnS_2_. (**a**) N-SnS_2_/NH_3_, (**b**) N-SnS_2_/Cl_2_, (**c**) N-SnS_2_/C_2_H_2_. The distance is Å.

Figure 6 shows the most stable structures of gas molecules on N-SnS_2_. For NH_3_ adsorption in Figure 6a, the structure of NH_3_ keeps intact in the adsorption process. The most stable structure for NH_3_ adsorption is obtained by the H atom of NH_3_ closing the N atom of N-SnS_2_, and the adsorption distance is 2.162 Å. The large adsorption distance indicates that the adsorption is physical adsorption. For Cl_2_ adsorption in Figure 6b, Cl atoms approach the surface of N with a single Cl atom. The adsorption distance reaches 2.915 Å. In addition, the chemical bond in Cl_2_ keeps intact in the adsorption process, only a slight elongation occurs in the Cl-Cl bond length, indicating that the adsorption is also weak physical adsorption. The adsorption structure of C_2_H_2_ is shown in Figure 6c. Its adsorption characteristics are similar to NH_3_, and the adsorption distance is 2.361 Å. The structure of C_2_H_2_ has not been damaged during the adsorption process.

The adsorption parameters of gases adsorbed N-SnS_2_ systems are listed in Table 3, including adsorption distance, adsorption energy, and charge transfer. It can be seen from the table that the adsorption energy of NH_3_ is −0.408 eV, and negative adsorption energy means that the reaction is exothermic and spontaneous. The charge transfer is 0.147 *e*, indicating a 0.147 *e* electron transfer from NH_3_ to N-SnS_2_. The small adsorption energy, long adsorption distance, and charge transfer confirm that the adsorption is physical adsorption. The adsorption energy of Cl_2_ is −0.245 eV, which is the lowest among the three gas adsorption, and its charge transfer is −0.136 *e*. The adsorption energy of C_2_H_2_ is −0.272 eV, which is the most moderate among the three gases. In total, 0.197 *e* electrons have been transferred to C_2_H_2_ from N-SnS_2_.

By comparing the adsorption of these three gases on B-SnS_2_ and N-SnS_2_, NH_3_ and C_2_H_2_ always give electrons to the substrate, whereas Cl_2_ always gains electrons. In addition, B-SnS_2_ has larger adsorption energy, larger charge transfer amount, and a shorter adsorption distance for the Cl_2_ adsorption system, indicating that the B-SnS_2_ monolayer has the most robust adsorption performance for Cl_2_ gas molecules. Based on the above analysis, it can be concluded that the modification of B enhances the adsorption activity of NH_3_, Cl_2_, and C_2_H_2_ to SnS_2_. The adsorption capacity of B-SnS_2_ to these gases is Cl_2_ > NH_3_ > C_2_H_2_.

**Figure 7:**
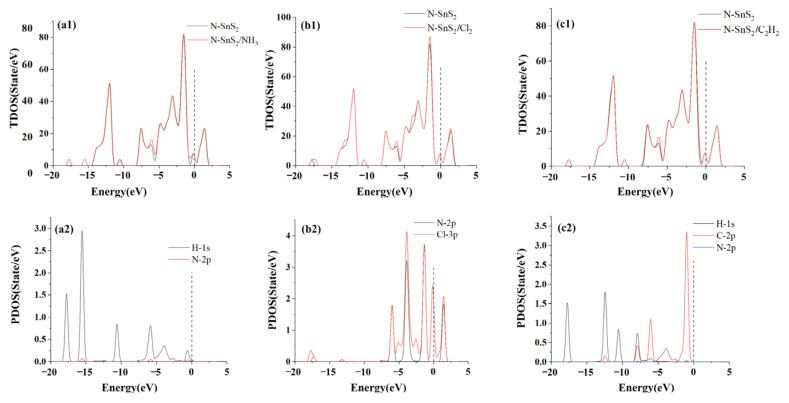
(**a1**) TDOS of N-SnS_2_ and N-SnS_2_/NH_3_, (**b1**) TDOS of N-SnS_2_ and N-SnS_2_/Cl_2_, (**c1**) TDOS of N-SnS_2_ and N-SnS_2_/C_2_H_2_, (**a2**) PDOS of N-SnS_2_/NH_3_, (**b2**) PDOS of N-SnS_2_/Cl_2_, (**c2**) PDOS of N-SnS_2_/C_2_H_2_.

As shown in Figure 7, TDOS and PDOS of all adsorbed gas systems were analyzed to further study the adsorption mechanism of the N-SnS_2_ system to the gas molecules, where dotted lines represent Fermi energy levels. TDOS and PDOS of N-SnS_2_ adsorption by NH_3_, Cl_2_, and C_2_H_2_ are shown in Figure 7(a1–c1) and Figure 7(a2–c2), respectively. For NH_3_ adsorption, the TDOS of the adsorption system increases a little near the Fermi level. It indicates that the conductivity of the adsorption system increases slightly. The interaction between N-2*p* and H-1*s* is fragile. After Cl_2_ adsorption, the atomic orbitals are strongly hybridized between the peaks of Cl-3*p* and N-2*p*. The TDOS of C_2_H_2_ nearly does not change after C_2_H_2_ adsorption on N-SnS_2_, and the corresponding interatomic orbital hybridization is also faint. Only the C-2*p* and H-1*s* peaks of the adsorption system overlap with the N atomic orbitals between −5.0 eV and −10.0 eV.

### 3.3. Analysis of Gas-Sensing Response

**Figure 8:**
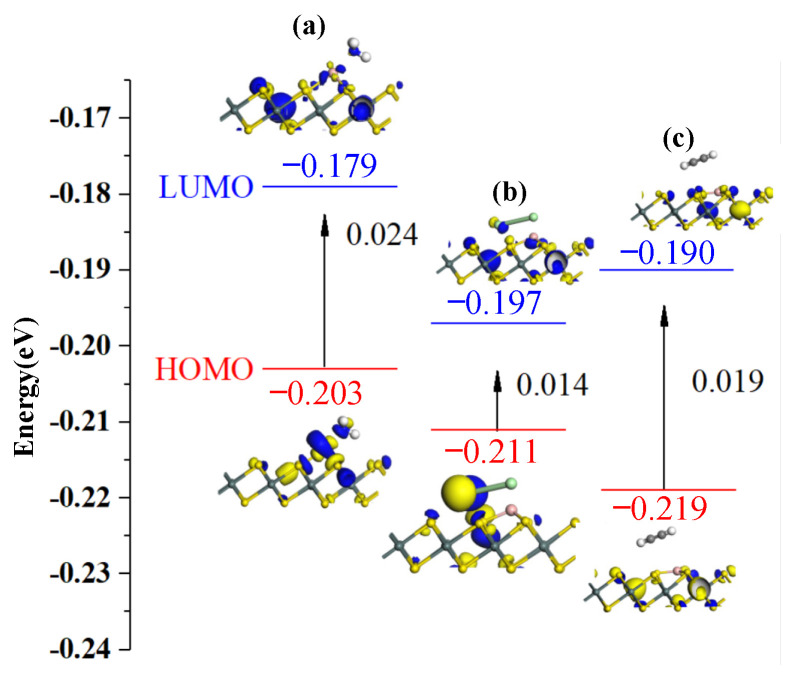
HOMO and LUMO of gas-adsorbed B-SnS_2_ systems: (**a**) B-SnS_2_/NH_3_, (**b**) B-SnS_2_/Cl_2_, (**c**) B-SnS_2_/C_2_H_2_.

The behavior of electrons in the adsorption process was analyzed by frontier molecular orbital theory. The HOMO and LUMO were obtained after NH_3_, Cl_2_, and C_2_H_2_ gas adsorption. It helps to explore gas sensors with selectivity and sensitivity. The HOMO and LUMO distributions before and after gas adsorption on B-SnS_2_ are shown in Figure 8, and the energy gap values are shown in Table 4. The adsorption charge transfer of Cl_2_ and NH_3_ molecules is significant. The HOMO and LUMO distributions are improved by gas adsorption, part of HOMO and LUMO transfer to Cl_2_ and NH_3_ molecules. The specific charge numbers corresponding to the analysis of the three gases are 0.254 *e*, −0.422 *e*, and 0.172 *e*, respectively. The charge transfer amount during the analysis of the adsorption process mainly comes from modified B atoms. Overall, the energy gap upon Cl_2_ and NH_3_ adsorption on the surface of B-SnS_2_ is bigger than that of C_2_H_2_. After adsorption, the energy gap value changes from 0.019 eV (B-SnS_2_) to 0.024 eV (B-SnS_2_/NH_3_), 0.014 eV (B-SnS_2_/Cl_2_), and 0.019 eV (B-SnS_2_/C_2_H_2_), respectively. A smaller energy gap indicates that the system’s conductivity improves, which is consistent with the previous DOS analysis.

**Figure 9:**
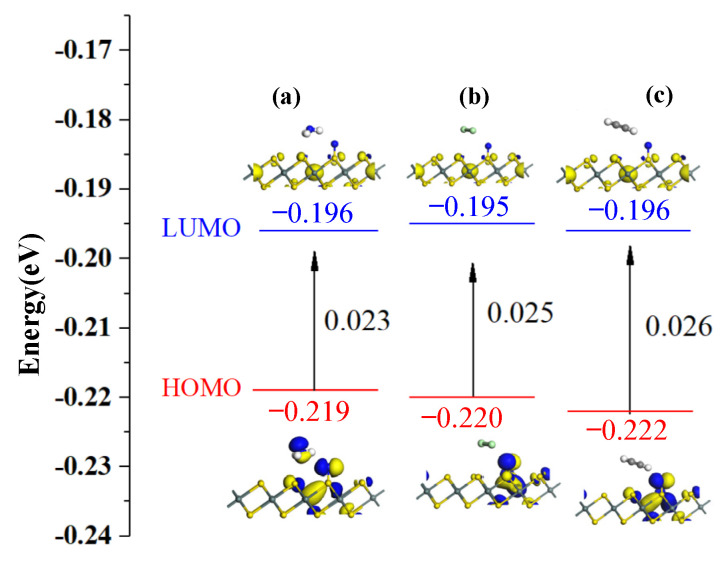
HOMO and LUMO of gas-adsorbed N-SnS_2_ systems: (**a**) N-SnS_2_/NH_3_, (**b**) N-SnS_2_/Cl_2_, (**c**) N-SnS_2_/C_2_H_2_.

The HOMO and LUMO distributions before and after gas adsorption on N-SnS_2_ are shown in Figure 9, and the energy gap values are shown in Table 5. HOMO is mainly distributed on N before N-SnS_2_ adsorbs gas, indicating that the N atom provides electrons as electron donors and is also the active site that provides adsorption sites for NH_3_, Cl_2_, and C_2_H_2_ gas. After adsorbing NH_3_, Cl_2_, and C_2_H_2_ gases, the HOMO becomes more concentrated on N, and the LUMO is nearly not located on gas molecules. From the HOMO and LUMO distribution of NH_3_ molecule adsorption, the E_g_ of N-SnS_2_/NH_3_ decreases to 0.023 eV. In addition, HOMO electrons are mainly located at H and N atoms, whereas LUMO electrons do not change significantly, which is consistent with the result obtained from TDOS and PDOS analysis. In contrast, the energy gap of N-SnS_2_/Cl_2_ is reduced to 0.025 eV, because HOMO electrons are mainly concentrated around N atoms, indicating that the adsorption of Cl_2_ molecules dramatically improves the conductivity and has better reactivity on N-SnS_2_ surfaces. In the C_2_H_2_ system, LUMO mainly concentrates around N atoms with long N-H bonds, reaching −0.196 eV. The increase in LUMO also reduces the energy gap of the system to 0.026 eV, resulting in a decrease in the conductivity of the system.

## 4. Conclusions

The adsorption of NH_3_, Cl_2_, and C_2_H_2_ molecules on B-SnS_2_ and N-SnS_2_ surfaces has been studied based on DFT calculation. The adsorption structure, charge transfer, DOS, and molecular orbital were analyzed to study the influence of B and N modification on the gas sensitivity of SnS_2_ monolayer to the gas molecules. Pristine SnS_2_ has low adsorption energy and a long adsorption distance for NH_3_, Cl_2_, and C_2_H_2_ gas molecules. Compared with the pristine SnS_2_, the adsorption capacity of the three gases on B-SnS_2_ and N-SnS_2_ is improved. The adsorption capacity of B-SnS_2_ to these gases is Cl_2_ > NH_3_ > C_2_H_2_. The adsorption capacity of N-SnS_2_ is NH_3_ > C_2_H_2_ > Cl_2_. TDOS and PDOS analysis results show that B-SnS_2_ has the strongest interaction with Cl_2_ and the weakest interaction with C_2_H_2_. Frontier molecular orbital analysis shows that the influence of gas molecules on the conductivity of the B-SnS_2_ adsorption system is NH_3_ > C_2_H_2_ > Cl_2_. The influence order of gas molecules on the conductivity of the N-SnS_2_ adsorption system is C_2_H_2_ > Cl_2_ > NH_3_. The results lay a theoretical foundation for developing B-SnS_2_ and N-SnS_2_ gas sensors for greenhouse gas detection.

## Figures and Tables

**Table 1:** Energy of HOMO, LUMO, and energy gap of B-SnS_2_ and N-SnS_2_.

Configuration	Structure	*E_HOMO_* (eV)	*E_LUMO_* (eV)	*E_g_* (eV)
B-SnS_2_	Figure 3a	−0.209	−0.190	0.019
N-SnS_2_	Figure 3b	−0.221	−0.196	0.025

**Table 2:** Adsorption parameters of gas molecules on B-SnS_2_.

System	Structure	*d* (Å)	*E*_ads_ (eV)	*Q*_T_ (e)
B-SnS_2_/NH_3_	Figure 4a	2.055	−1.735	0.254
B-SnS_2_/Cl_2_	Figure 4b	1.776	−2.204	−0.422
B-SnS_2_/C_2_H_2_	Figure 4c	2.531	−0.272	0.172

**Table 3:** Adsorption parameters of gas molecules on N-SnS_2_.

System	Structure	*d* (Å)	*E*_ads_ (eV)	*Q*_T_ (e)
N-SnS_2_/NH_3_	Figure 6a	2.162	−0.408	0.147
N-SnS_2_/Cl_2_	Figure 6b	2.915	−0.245	−0.136
N-SnS_2_/C_2_H_2_	Figure 6c	2.361	−0.272	−0.197

**Table 4:** Energy of HOMO, LUMO, and energy gap of B-SnS_2_ and adsorption systems.

Configuration	Structure	*E_HOMO_* (eV)	*E_LUMO_* (eV)	*E_g_* (eV)
B-SnS_2_/NH_3_	Figure 8a	−0.203	−0.179	0.024
B-SnS_2_/Cl_2_	Figure 8b	−0.211	−0.197	0.014
B-SnS_2_/C_2_H_2_	Figure 8c	−0.209	−0.190	0.019

**Table 5:** Energy of HOMO, LUMO, and energy gap of N-SnS_2_ and adsorption systems.

Configuration	Structure	*E_HOMO_* (eV)	*E_LUMO_* (eV)	*E_g_* (eV)
N-SnS_2_/NH_3_ N-SnS_2_/Cl_2_	Figure 9a Figure 9b	−0.219 −0.220	−0.196 −0.195	0.023 0.025
N-SnS_2_/C_2_H_2_	Figure 9c	−0.222	−0.196	0.026
